# Protective role of the deSUMOylating enzyme SENP3 in myocardial ischemia-reperfusion injury

**DOI:** 10.1371/journal.pone.0213331

**Published:** 2019-04-11

**Authors:** Nadiia Rawlings, Laura Lee, Yasuko Nakamura, Kevin A. Wilkinson, Jeremy M. Henley

**Affiliations:** School of Biochemistry, Centre for Synaptic Plasticity, Biomedical Sciences Building, University of Bristol, University Walk, Bristol, United Kingdom; University of Cincinnati College of Medicine, UNITED STATES

## Abstract

Interruption of blood supply to the heart is a leading cause of death and disability. However, the molecular events that occur during heart ischemia, and how these changes prime consequent cell death upon reperfusion, are poorly understood. Protein SUMOylation is a post-translational modification that has been strongly implicated in the protection of cells against a variety of stressors, including ischemia-reperfusion. In particular, the SUMO2/3-specific protease SENP3 has emerged as an important determinant of cell survival after ischemic infarct. Here, we used the Langendorff perfusion model to examine changes in the levels and localisation of SUMOylated target proteins and SENP3 in whole heart. We observed a 50% loss of SENP3 from the cytosolic fraction of hearts after preconditioning, a 90% loss after ischemia and an 80% loss after ischemia-reperfusion. To examine these effects further, we performed ischemia and ischemia-reperfusion experiments in the cardiomyocyte H9C2 cell line. Similar to whole hearts, ischemia induced a decrease in cytosolic SENP3. Furthermore, shRNA-mediated knockdown of SENP3 led to an increase in the rate of cell death upon reperfusion. Together, our results indicate that cardiac ischemia dramatically alter levels of SENP3 and suggest that this may a mechanism to promote cell survival after ischemia-reperfusion in heart.

## Introduction

Standard clinical treatment after a heart attack is to restore blood supply as soon as possible in order to limit infarct size and reduce mortality. Paradoxically, however, this can cause oxidative damage, referred to as reperfusion injury, which leads to cardiomyocyte cell death and contributes to reduced cardiac output [1]. An important goal in the field is to identify the cellular changes that occur during ischemia and subsequent reperfusion, with a view to determining how intervening in these pathways may promote cell viability after ischemic insult.

Particularly relevant is the phenomenon of ischemic preconditioning, whereby tissues exposed to repeated short bursts of ischemia protect the tissue against the effects of prolonged ischemia. How preconditioning leads to cellular protection is poorly understood so defining the pathways involved and designing strategies to potentiate them to promote cellular survival after ischemia represent important goals. Important clues come from animals that hibernate because they have very low rates of blood flow, so tissues endure prolonged hypoxia but emerge from hibernation torpor undamaged. It has emerged that one protective mechanism is massively increased levels of protein post-translational modification of lysine residues by small ubiquitin-like modifier (SUMO) during torpor [2, 3].

There are three ~11 kDa SUMO paralogues (SUMO-1-3) in mammals that are conjugated to substrates, generally within a consensus motif [4, 5]. While the functional consequences of SUMOylation vary depending on the substrate, the underlying principle is that it alters inter- and/or intramolecular interactions of substrate proteins to change their localisation, stability, and/or activity [6].

SUMOylation is dynamic and SUMO can be removed from proteins by SUMO proteases (SPs). Nine mammalian SPs have been identified but their specific targets and physiological roles are poorly characterized and how SP activity is regulated to control substrate deSUMOylation is largely unknown (reviewed in [7–9]). The largest family of SPs are the sentrin-specific proteases (SENPs). Generally, SENP1 and SENP2 mature pre-SUMOs and deconjugate SUMO1 and SUMO2/3, SENP3 and SENP5 preferentially deconjugate SUMO2/3, and SENP6 and SENP7 can edit polySUMO2/3 chains [10, 11].

Significant changes in SUMO pathway components occur during ischemia in the human heart [12]. Moreover, over-expression of SUMO enhances cardiac function in mice with heart failure and increases contractility in isolated cardiomyocytes [13]. Interestingly, recent data also suggests a role for SENP3 in determining cardiomyocyte survival after ischemia-reperfusion, however whether SENP3 promotes cell survival or cell death remains controversial. It has been reported that SENP3 levels are upregulated in heart tissue in response to ischemia-reperfusion, and that knockdown of SENP3 *in vivo* reduces infarct size and improves cardiac function [14]. Similarly, in the cardiomyocyte H9C2 cell line, there is an increase in SENP3 levels in response to ischemia-reperfusion but, in contrast, knockdown of SENP3 promoted apoptosis after ischemia-reperfusion [15].

To directly address this controversy and characterise the molecular events that occur during ischemia and reperfusion, we investigated how SUMOylation and levels of SENP3 are altered by ischemia and ischemia-reperfusion in whole heart. To investigate the molecular mechanisms that underlie cell protection afforded by ischemic preconditioning, we also examined hearts which were subjected to 3 short bursts of ischemia-reperfusion prior to the extended ischemic insult. Cytosolic levels of SENP3 were dramatically reduced by ischemia and ischemia-reperfusion. Furthermore, knockdown of SENP3 in the cardiomyocyte H9C2 cell line demonstrated that loss of SENP3 increases the rate of cell death in response to ischemia-reperfusion. Together, these data suggest an important role for SENP3-mediated deSUMOylation in promoting cardiomyocyte survival after ischemic insult.

## Materials and methods

### Heart perfusion

All procedures were performed in accordance with Schedule 1 Guidance on the Operation of the Animals (Scientific Procedures) Act 1986 and are described previously [16]. In brief, male Wistar rats (250-275g) were anaesthetised with isofluorane and killed by cervical dislocation. Hearts were rapidly cannulated via the aorta *in vivo*, then quickly removed and placed into 37°C buffered Krebs-Henseleit solution containing in (mM): NaCl 118, NaHCO_3_ 25, KCl 4.8, KH_2_PO_4_ 1.2, MgSO_4_ 1.2, glucose 11 and CaCl_2_ 1.2, gassed with 5% CO_2_ at 37°C (pH 7.4). Hearts were perfused at 12 ml min^−1^ in the Langendorff mode with in-line filter using Krebs-Henseleit buffer. No electrical stimulation was applied. Left ventricular developed pressure (LVDP) was monitored with a water-filled balloon inserted into the left ventricle. Initial end diastolic pressure (EDP) was set to give an initial value of 4–5 mm Hg. All hearts were allowed an equilibration period of at least 20 min before the time for each group was started. Control group (CTL) was perfused for 45 min. Global normothermic ischemia was induced by switching off the pump and submersing the heart in buffer kept at 37°C. In all groups the ischemic period was 30 min. The preconditioned-ischemic H9C2group was subjected to three cycles of 2 min of global ischemia interspersed with 3 min reperfusion prior to 30 min global normothermic ischemia. In the ischemia-reperfusion group, after 30 min of global ischemia buffer flow was restored for the next 2 hours.

### Subcellular fractionation of left ventricular tissue

Isolation of subcellular fractions from rat left ventricular tissue was adapted from a previously published protocol [17]. Left ventricular tissue was immersed in 6 ml ice cold isolation buffer (300 mM sucrose, 3 mM EGTA, 10 mM Tris-HCl, pH 7.1) supplemented with 20 mM NEM, 1x complete protease inhibitors (Roche) and 1x phosphatase inhibitors (Roche). Tissue was rapidly chopped with scissors into fine pieces before homogenisation using a Polytron tissue disruptor (Kinematica) at 10,000rpm with 2 bursts of 5 seconds followed by 1 burst of 10 seconds. Tissue homogenate was diluted to 20 ml total volume with isolation buffer supplemented with 1x complete protease inhibitors and further homogenised by hand for 2 minutes using a glass Potter homogeniser and Teflon pestle. A small volume of homogenate was stored at -80°C as whole homogenate. The rest of the homogenate was centrifuged at 7500g for 7 minutes and the soluble fraction (supernatant) stored at -80°C as a cytosol-containing fraction. The pellet was rinsed twice with 5 ml isolation buffer, resuspended in 20mL isolation buffer and further hand-homogenised for 2 minutes. The homogenate was then centrifuged at 600g for 10 minutes and the pellet resuspended in isolation buffer and stored at -80°C as a nucleus-containing fraction. The supernatant was centrifuged at 7000g for 10 minutes to yield a crude mitochondrial pellet, which was resuspended in isolation buffer and stored at -80°C. Proteins from soluble cytosol-containing fraction were precipitated with acetone for at least 1h at -20°C, spun down and resuspended in isolation buffer. All fractions were assayed for protein concentration using a standard BCA assay protocol prior to Western blotting.

### SENP3 knockdown

An shRNA sequence targeting rat SENP3 (target sequence TATGGACAGAACTGGCTCAATGACCAGGT), or a non-targeting (‘scrambled’) control (target sequence GCACTACCAGAGCTAACTCAGATAGTACT) were cloned into a modified pXLG viral vector under the control of a U6 promoter. Viral particles were produced in HEK293T cells using the helper vectors pMD2.G and p8.91 as described previously [18], and virus-containing supernatant used to transduce H9C2 cells.

### Lactate dehydrogenase (LDH) assay

Medium was collected from experimental wells, placed on ice, spun down at 1000g for 1 min and tested for LDH within 30 min of collection. The LDH cytotoxicity detection kit was purchased from Clontech and used according to the manufacturer’s protocol. Briefly, one volume each of tested sample and working solution were mixed in a 96 well plate, and incubated for 30 minutes at RT, protected from light. The absorbance was then read in a spectrophotometer at 490nm and 620nm. LDH activity in each well was calculated by subtracting the 620nm reading (plastic plate background) from the 490nm reading. Each experiment contained the following controls: background control (medium only)–subtracted from all values as a background reading; high control—measures the maximum LDH activity that can be released from the 100% dead cells in response to medium containing 1% Triton X-100. Values of % dead cells in experimental wells were calculated with respect to the 100% dead cells value from Triton X-100 treated cells. Importantly, for these experiments, media lacking phenol red and sodium pyruvate was used as both components interfere with LDH readings.

### H9C2 cell culture and ischemia-reperfusion protocol

H9C2 (2–1) cells were purchased from Merck and cultured in media (FG 0435, Biochrome) containing 10% FBS Superior (S0615, Biochrome) and 0.05% penicillin/streptomycin for 5 days. For LDH measurements 20x10^3^ cells were plated per well in a 6-well plate; for OGD experiments– 120x10^3^ cells per 100 mm dish. 2 days before experiments 10% FBS-containing medium was replaced with medium containing 1% horse serum (New Zealand origin, Invitrogen) and 0.05% penicillin/streptomycin.

For ischemia only or ischemia-reperfusion experiments all cells were washed with PBS and changed from phenol red-containing culture medium to transparent media (Gibco, A1443001) containing 1% horse serum and 4.5g/L glucose for 1h. All time-matched controls had their glucose-containing media replaced with the same glucose-containing media the same amount of times as their respective ischemia or ischemia-reperfusion conditions. Ischemia was achieved by placing cells in the MACS-VA500 anaerobic workstation (Don Whitley Scientific limited) supplemented with 95% N_2_ and 5% CO_2_ at 37°C. Once in the anaerobic workstation, cells were washed with deoxygenated PBS and then placed in deoxygenated medium containing 1% horse serum and lacking glucose for the specified time. In order to achieve quick reperfusion, at the end of ischemia, cells were quickly changed back into oxygen and glucose-containing media inside the chamber and plates were placed back in standard cell culture incubators (5% CO_2_, 37°C).

### H9C2 subcellular fractionation

Before collection all cells were washed 4 times with ice-cold PBS to remove horse serum-containing media. Subcellular fractionation was then performed based on a previously published protocol [19] with some modifications. Briefly, cells from a 100 mm dish were collected in 1 ml of prechilled lysis buffer A (100 mM NaCl in 50 mM HEPES containing 25 μg/ml digitonin, protease and phosphatase inhibitor cocktails and 20 mM NEM). Cells were then hand homogenized in a glass homogenizer on ice and spun for 30 min at 16000g at 4°C. Supernatant was collected as a cytosolic sample, and pellets were collected as a membrane/nuclear fraction. The membrane/nuclear-containing pellet was then resuspended and sonicated in lysis buffer B (100 mM NaCl in 50 mM HEPES buffer, containing 5 mg /ml sodium deoxycholate, 1% Triton-X, 0.5% SDS, protease and phosphatase inhibitor cocktails and 20 mM NEM). Since the cytosolic fraction was too dilute, proteins were precipitated in 5 volumes of prechilled acetone for 2h at -20°C, then spun down for 30 min at 3000g at 4°C. The pellet containing the cytosolic sample was then resuspended and sonicated in lysis buffer B. All samples were evaluated for protein concentration by BCA assay (Thermo Fisher).

### Immunoblotting

Samples were resolved by SDS-PAGE (7.5–15% gels), transferred to Immobilon-P membranes (Millipore Inc.) and immunoblotted as indicated. Primary antibodies used were: SUMO-1 and SUMO-2/3 (sheep, a gift from Ron Hay (University of Dundee)), VDAC (Cell Signalling, D73D12), GAPDH (Sigma, G9295), SENP3 (Cell Signaling, D20A10), Lamin (Santa Cruz, sc-6216), and cleaved caspase 3 (Cell Signaling, 5A1E). Total protein on membranes was stained using REVERT total protein stain (LI-COR). Immune complexes were detected using HRP-conjugated secondary antibodies (Sigma) followed by enhanced chemiluminescence (Thermo Scientific Pierce) using a LI-COR Odyssey machine or x-ray films.

### Statistics

All statistical analyses are specified in the Figure legends.

## Results

### Characterisation of haemodynamic properties of perfused hearts

To examine the molecular changes that occur during ischemia, ischemia-reperfusion and ischemic-preconditioning, we used the Langendorff perfused isolated rat heart model. We chose to evaluate 4 experimental groups (S1 Fig).

- **Control**, 20 min of stabilization perfusion and 50 min perfusion with Krebs-Henseleit buffer (KHB).
- **Preconditioning and ischemia (PCI)**, 20 min of stabilization perfusion followed by 3 cycles of 2 min ischemia and 3 min reperfusion (15 min of preconditioning) and then 30 min of ischemia.
- **Ischemia**, 20 min of stabilization perfusion, 20 min perfusion and 30 min of global ischemia.
- **Ischemia + reperfusion (I/R)**, 20 min of stabilization perfusion, 20 min perfusion, 30 min global ischemia and 2h of reperfusion.

We first examined several haemodynamic parameters to validate the model. These were left ventricular developed pressure (LVDP), end-diastolic pressure (EDP), heart rate, time to ischemic contracture and maximal contracture. As shown in Table 1, in preconditioned hearts the time to ischemic contracture was significantly shorter than in ischemic hearts, indicating preconditioning occurred in our hearts consistent with previous observations [20]. Moreover, since time to ischemic contracture was the same in the ischemia and I/R groups, we conclude that there was no unintended preconditioning in the I/R group and that any differences between these groups can be attributed to ischemia-reperfusion injury.

**Table 1:** Haemodynamic parameters for Langendorff perfused hearts.

	Control n = 6	Preconditioned- Ischemia n = 5	Ischemia n = 6	Ischemia / reperfusion n = 6
*20 min point*				
LVPD (mm Hg)	79.7±14.4	81.8±3.6	73.3±8.4	76.0±5.6
EDP (mm Hg)	8.4±0.8	8.0±1.0	11.3±1.2	7.6±0.9
HR (bpm)	321±7	308±13	310±13	338±19
*Ischemia*				
Time to IC (min)	-	7.7±1.2**	13.5±0.8	12.8±0.5
Max IC (mm Hg)	-	68.4±10.0	54.6±7.0	42.4±3.9

All values are given as mean ± SEM. Data are shown for hearts used for further sample collection and Western blot sampling. LVPD–left ventricular developed pressure; EDP–end-diastolic pressure; HR–heart rate; IC–ischemic contracture. Note that because we collected our heart samples for biochemical analyses at the end of each described stage, we were unable measure recovery of LVPD for the preconditioned group since there was no reperfusion after ischemia.

** Significant difference between PCI group and Isc and I/R groups (p<0.01). One-way ANOVA with Bonferroni’s multiple comparison test was used.

### Subcellular fractionation of whole hearts

To profile changes in SUMOylation and SENP3, we prepared whole tissue homogenates from hearts that had undergone the Langendorff protocol as well as separate cytosolic, nuclear and mitochondrial fractions. Following trial experiments to optimise the fraction separation and retain mitochondria-associated proteins we used a relatively gentle mitochondrial enrichment fractionation protocol (see methods).

### Protein SUMOylation in perfused heart

**Fig 1:**
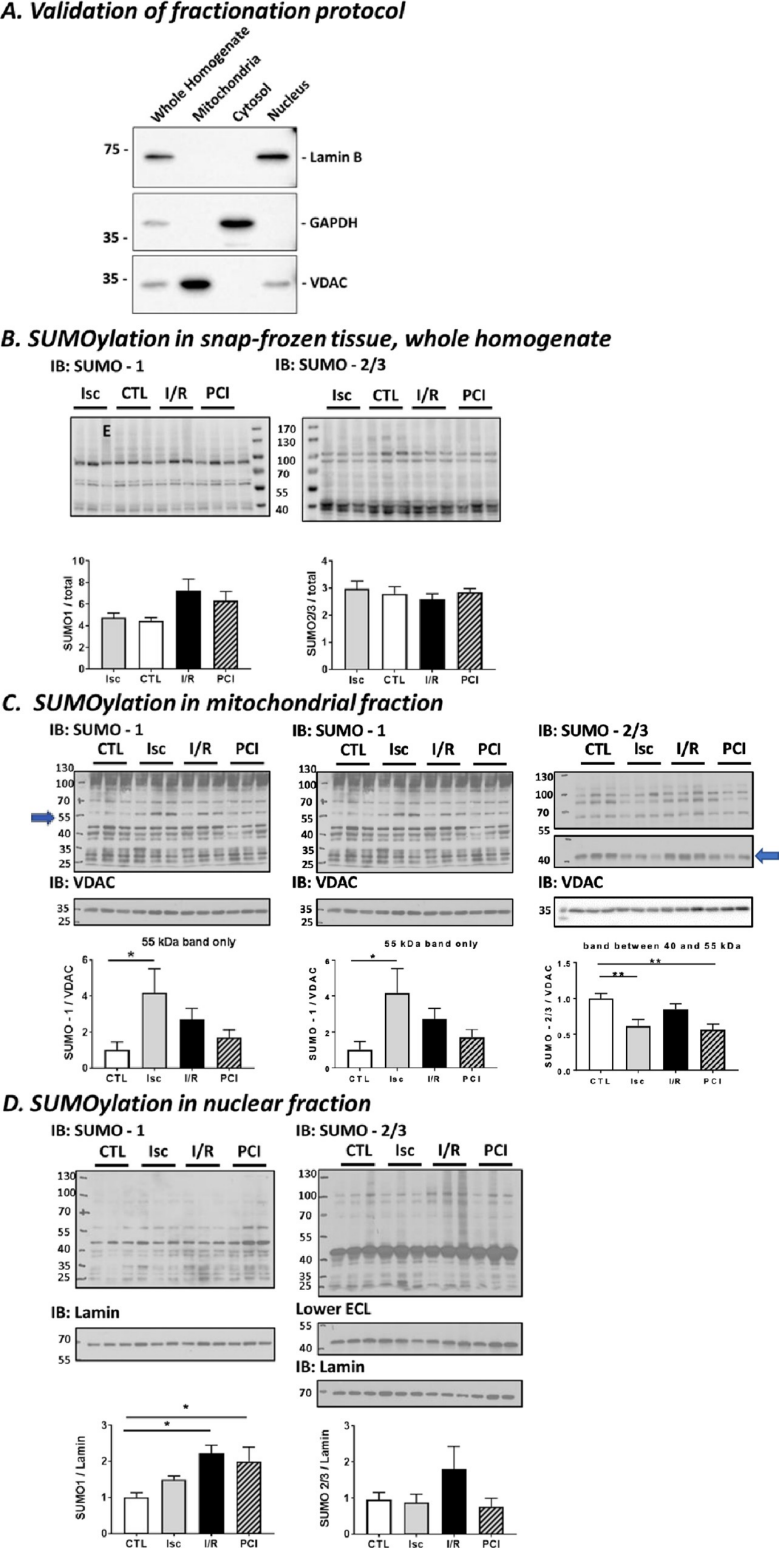
Total protein SUMOylation in Langendorff perfused rat heart following preconditioning-ischemia (PCI), ischemia (Isc) only and ischemia followed by reperfusion (I/R). Representative SUMO-1 (left) or SUMO-2/3 (right) Western blots of snap frozen left ventricular tissue fractions from rat heart following Langendorff perfusion. Blots show representative blots samples prepared from at least 3 different Langendorff perfused hearts. (**A**) Validation of the fractionation protocol used. Equal amounts of whole homogenate or mitochondrial, cytosolic or nuclear fraction from left ventricular rat heart tissue were Western blotted as indicated. Lamin was used as a nuclear marker, GAPDH as a cytosolic marker and VDAC as a mitochondrial marker. (**B**) Whole homogenate. In the left-hand Western blot shown in panel A “**E**” denotes a sample that was excluded from analysis based on different haemodynamic parameters from the rest of the ischemic group. (**C**) Mitochondria-enriched fraction. Histograms show quantification of the changes in a specific ~ 55kDa band in the SUMO-1 blot (left) and a ~ 45kDa band in the SUMO-2/3 blot (right) that were markedly altered between conditions. (**D**) Nuclear fraction. Histograms show quantification of the changes in total nuclear protein SUMOylation levels. Total protein stain with Ponceau, VDAC or Lamin antibodies, were used as a loading control for whole homogenate samples, mitochondrial or nuclear factions, respectively. Quantitative analysis was performed using ordinary one-way ANOVA with Sidak’s correction for multiple comparisons with a pooled variance. **N = 6 for CTL, PCI and I/R; N = 5 for Isc group**. Data presented as mean ± SEM. *p<0.05; ** p<0.01.

Having validated the experimental system, we next measured levels of SUMO1- and SUMO2/3-ylation in total homogenates and mitochondrial and nuclear fractions from the perfused hearts. The purity of the subcellular fractions was validated using specific mitochondrial, cytosolic and nuclear marker proteins, confirming the high levels of separation of each fraction (Fig 1A). No significant changes were detected in total SUMO-immunoreactivity in whole homogenates (Fig 1B). Similarly, we did not detect any overall changes in protein SUMOylation in the mitochondrial fraction (Fig 1C). It should be noted, however, that these global protein assays do not exclude the possibility of changes in the SUMOylation status of individual or subsets of mitochondrial proteins.

Indeed, an approximately 55kDa SUMO-1-ylated species was significantly upregulated in the mitochondrial fraction under ischemia, compared to pre-ischemia, but was not upregulated during ischemia in tissue that had been preconditioned or after reperfusion (indicated by arrow in left hand blot Fig 1C). Similarly, while there were no global alterations in SUMO-2/3 conjugation, the SUMO-2/3-ylation of an individual mitochondrial substrate at ~45kDa was reduced by preconditioning and ischemia (indicated by arrow in right hand blot Fig 1C).

Likewise, we did not detect changes in total nuclear protein SUMO-2/3-ylation in any of the conditions. In contrast, however, we observed robust changes in SUMO-1-ylation of nuclear proteins, with significantly increased levels in preconditioned and I/R conditions (Fig 1D). Thus, while no striking differences in the overall levels of SUMOylated proteins in whole homogenate or mitochondria were detected, changes were observed for individual SUMO-1 and SUMO-2/3 substrate proteins, and for total SUMO-1-ylation in the nucleus.

### Changes in SENP3 during ischemia and reperfusion

**Fig 2:**
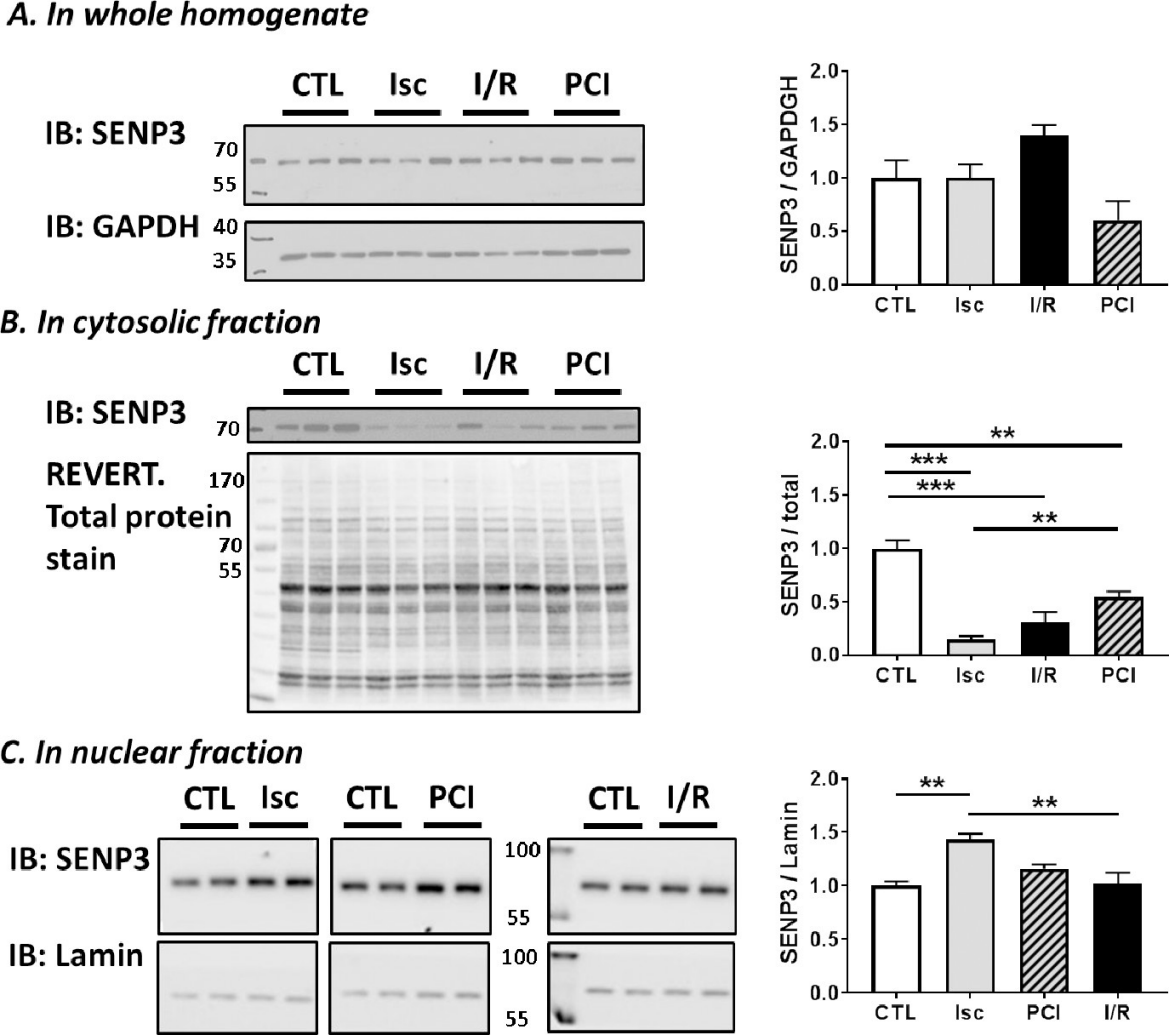
SENP3 levels in different subcellular fractions of Langendorff perfused rat heart. Representative blots of 2–3 samples per condition from the left ventricular tissue of different Langendorff perfused hearts Western blotted for SENP3. (**A**) Whole homogenate, showing no change in total SENP3. (**B**) Cytosolic fraction, demonstrating reduced SENP3 in all ischemia conditions. (**C**) nuclear fraction, showing an increase of SENP3 during ischemia. Total protein stain (using REVERT total protein stain), GAPDH or Lamin antibodies were used as loading controls and are shown under each blot. Quantitative analysis was performed with an ordinary one-way ANOVA with Sidak’s correction for multiple comparisons with a pooled variance. **N = 6 for CTL, PCI and I/R; N = 5 for Isc group**. Data presented as mean ± SEM. *p<0.05; ** p<0.01, *** p<0.001.

The extent and duration of substrate SUMOylation is determined by the balance between conjugation and SUMO-protease-mediated deconjugation. SENP3 is one of the best characterised SUMO-proteases that preferentially removes SUMO-2/3 from target proteins [9]. Importantly, SENP3 has recently emerged as a key determinant of cell survival after ischemic stress [6, 21, 22]. We therefore tested the effects of preconditioning, ischemia and reperfusion on SENP3 levels in total homogenate, cytosol and nuclear fractions of Langendorff perfused whole heart (Fig 2). There were no significant changes in the total levels of SENP3 between the control, preconditioning, ischemia and I/R conditions in whole homogenates (Fig 2A). In the cytosolic compartment, however, SENP3 levels were significantly decreased in the preconditioned, ischemia, and I/R groups. Moreover, this loss of SENP3 levels in cytosol during ischemia was significantly attenuated by prior preconditioning (Fig 2B). Correlating with these data, we observed an increase in SENP3 in the nuclear fraction in the ischemia group, the condition in which there was the biggest decrease in cytosolic SENP3 (Fig 2C). Taken together, these data suggest that ischemia leads to a translocation of SENP3 from the cytosol to the nucleus, and that the magnitude of this translocation can be reduced by preconditioning prior to the insult.

### Ischemia and ischemia-reperfusion in H9C2 cells

We next wondered how SENP3 levels affect cell survival in cardiomyocytes in response to ischemia and ischemia-reperfusion. To do this, we used the well characterised rat ventricle origin cardiomyoblast H9C2 cell line [23], since this model system allowed us to genetically manipulate SENP3 levels prior to ischemic insult.

**Fig 3:**
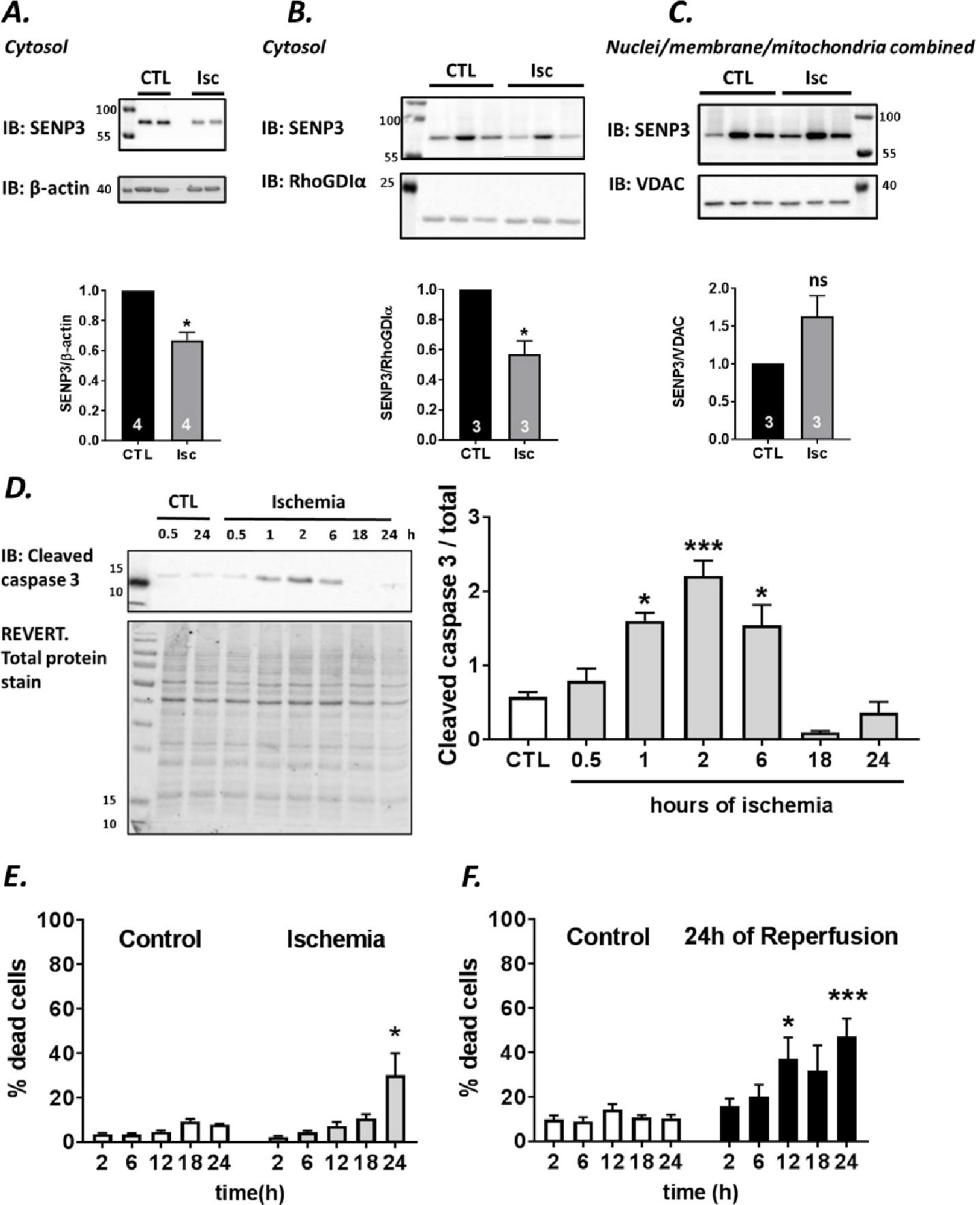
Ischemia and ischemia-reperfusion in H9C2 cells. (**A, B**) Effects of ischemia on cytosolic SENP3 levels in two separate sets of experiments. SENP3 levels were normalized to β-actin (**A**) or RhoGDI (**B**). (**C**) SENP3 levels in a pooled membrane/nuclear fraction, normalized to VDAC. Raw data were analysed using Students t-test to compare control to ischemia conditions. Data presented as normalized to control values, mean ± SEM. *p<0.05. (**D**) Activation of cleaved caspase 3 monitored by the ~17kDa cleavage fragment shows bell-shaped response with peak following 2h of ischemia. Proteins normalized to total protein labelled with REVERT stain. Representative blot and quantitative analysis using ordinary one-way ANOVA with Sidak’s correction for multiple comparisons with a pooled variance. Data presented as mean ± SEM. *p<0.05; **** p<0.001. (**E**) Profile of LDH release in ischemia experiments, N = 3. (**F**) Profile of LDH release assay in experiments where 2-24h ischemia was followed by 24h of reperfusion, N = 3 experiments. For (**E**) and (**F**), Students t-test comparing control to ischemia at each time point. Data presented as mean ± SEM. *p<0.05; *** p<0.001.

First, we confirmed that H9C2 cells respond to ischemic insult in a similar way to whole heart. In agreement with Langendorff perfused whole heart data, there was a significant decrease in cytosolic SENP3 within the first 30 min of ischemia using two different marker proteins, β-actin and RhoGDIα, to normalise our results (Fig 3A and 3B). Interestingly, in the combined nuclear/mitochondia membrane fraction SENP3 showed a strong, but non-significant, trend to increase. We attribute the lack of significance to the already very high and somewhat variable levels of nuclear SENP3 in these cells. Because of this the translocation of comparatively small amounts of cytosolic SENP3 to the nucleus was not significant (Fig 3C). Nonetheless, these results confirm that this cell line represents a useful model system for examining the role of SENP3 in the cellular response to ischemic stress.

Therefore, we profiled the time course of markers of apoptosis and cell death in H9C2 cells in response to ischemia to identify appropriate timepoints to examine the role of SENP3 in cell survival. We subjected cells to varying periods of ischemia and assayed the initiation of apoptosis using cleavage of the executioner caspase 3 [24] (Fig 3D). Caspase 3 activation showed a bell-shaped response plotted against time of ischemia, with the peak at 2h and a drop below control levels at around 18h. Additionally, we assayed cell death by measuring LDH release into the culture medium. Interestingly, H9C2 cells are very resilient to ischemia and ischemia-repurfusion challenges, requiring 24h of ischemia alone (Fig 3E) or minimum 12h of ischemia followed by 24h of reperfusion (Fig 3F) to induce significant levels of cell death.

### Effects of SENP3 knockdown on H9C2 cell response to ischemic stress

**Fig 4:**
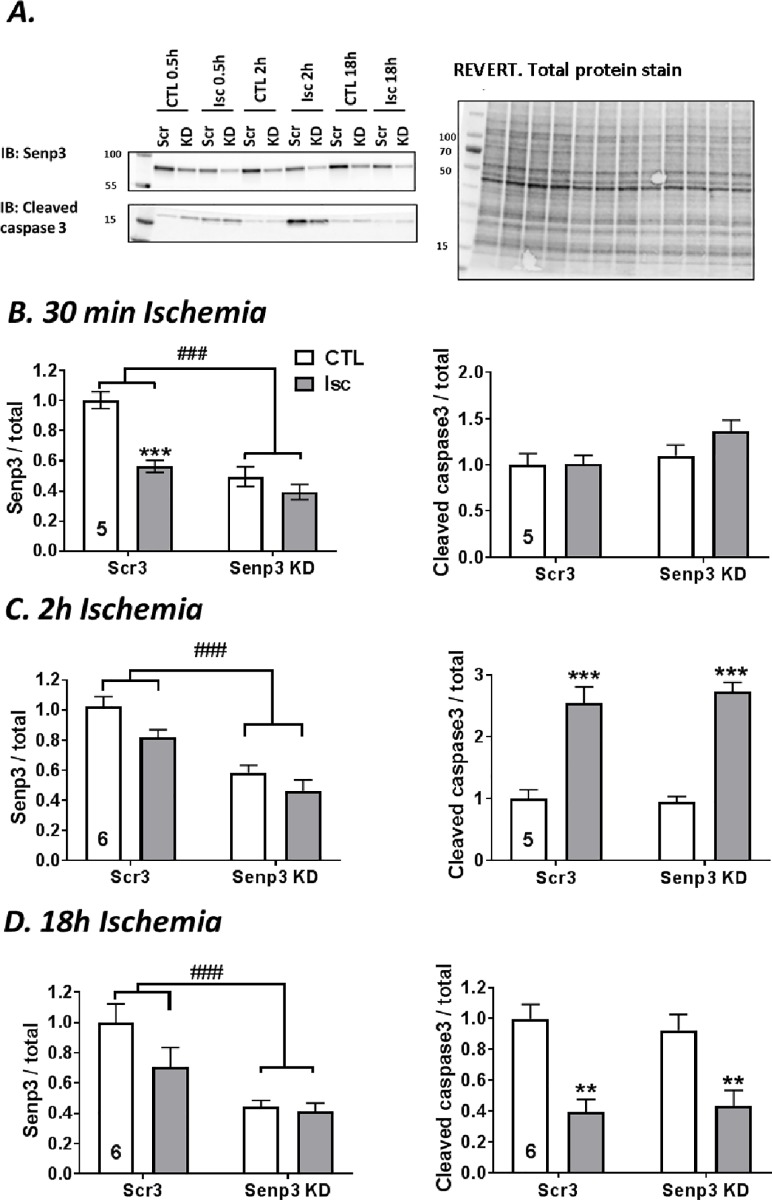
Effects of 30 min, 2h and 18h ischemia on SENP3 levels and caspase 3 activation in control and SENP3 knockdown H9C2 cells. (**A**) Representative Western blots showing the effects of SENP3 knockdown. Scr3 = control, scrambled non-targeting shRNA, Senp3 KD = shRNA targeting SENP3. The righthand panel shows total protein levels visualised using REVERT protein stain. Histograms show quantification of levels of cytosolic SENP3 and cleaved caspase 3 following (**B**) 30 min of ischemia, (**C**) 2h of ischemia, (**D**) 18h of ischemia. Number of experiments indicated in each graph. Quantitative analysis using ordinary two-way ANOVA test with Sidak’s correction for multiple comparisons. Data presented as mean ± SEM. * show p values for effect of ischemia; # show p values for effect of SENP3 KD.

Having established conditions to monitor ischemia-induced cell death in H2C9 cells we next investigated the effects partial SENP3 knockdown. We infected H9C2 cells with lentivirus expressing either a control scrambled non-targeting shRNA or an shRNA targeting SENP3, which reduced levels of SENP3 by ~50%. 14 DIV after infection cells were subjected to 30 min, 2h or 18h of ischemia (Fig 4).

In the scrambled shRNA control condition, 30 min ischemia reduced cytosolic SENP3 by 50%, about the same reduction as achieved by infection with SENP3 shRNA. Moreover, ischemia did not further decrease in SENP3 levels in the SENP3 knockdown cells. Surprisingly, however, 2h and 18h periods of ischemia did not significantly reduce SENP3 in the control cells, nor was there and additional reduction in SENP3 levels in the SENP3 knockdown cells (Fig 4A). These results indicate that cytosolic levels of SENP3 are rapidly reduced during ischemia, but that levels recover during prolonged ischemia.

To examine the link between SENP3 levels and initiation of apoptosis, we tested the effect of SENP3 knockdown on ischemia-induced cleavage of caspase 3. Consistent with the data shown in Fig 3D, there was a significant increase in cleaved caspase 3 after 2h ischemia, which was not affected by SENP3 knockdown (Fig 4B and 4C). In contrast, cleaved caspase 3 was significantly reduced after 18h ischemia in both control cells, containing scrambled non-targeting shRNA, and SENP3 knockdown cells (Fig 4D). Again, this finding correlates with the drop of activated cleaved caspase 3 found in uninfected H9C2 cells after 18h of ischemia (Fig 3D).

### SENP3 knockdown increases the rate of cell death during ischemia-reperfusion in H9C2 cells

**Fig 5:**
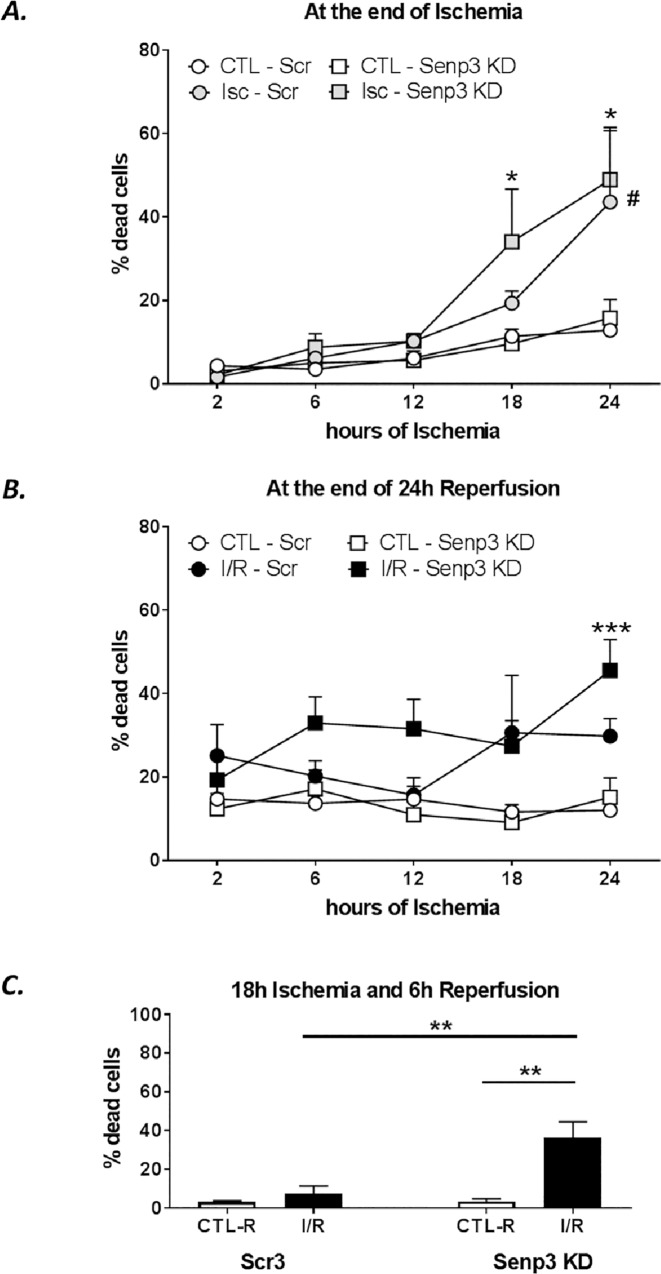
SENP3 knockdown in H9C2 cells increases susceptibility to ischemic cell death. Time course of ischemia-induced cell death measured by LDH assays in control and SENP3 knockdown H9C2 cells. Scrambled shRNA control, round symbols, SENP3 KD, square symbols. (**A**) LDH release after indicated times of control (white symbols) and ischemia (grey symbols), N = 3–4 independent experiments. Data presented as mean ± SEM. Quantitative analysis using ordinary two-way ANOVA with Sidak’s correction for multiple comparisons with a pooled variance. * p<0.05 for SENP3 KD group, # p<0.05 for scrambled group, both control vs ischemia. No differences were detected between control and SENP3 KD cells. (**B**) LDH release after indicated times of control (white symbols) and ischemia followed by 24h of reperfusion (black), N = 3–4 independent experiments. Data presented as mean ± SEM. Quantitative analysis using ordinary two-way ANOVA with Sidak’s correction for multiple comparisons with a pooled variance. *** p<0.001 for SENP3 KD group, control vs ischemia-reperfusion. No difference between scrambled or KD cells has been observed. (**C**) LDH release after 18h ischemia followed by 6h reperfusion, showing increased rate of cells death for SENP3 KD group. N = 3 experiments. Data presented as mean ± SEM. Quantitative analysis using ordinary two-way ANOVA test with Sidak’s correction for multiple comparisons with a pooled variance, ** p<0.01.

We next examined how SENP3 affects cell death in response to ischemia-reperfusion by measuring LDH release. SENP3 knockdown did not alter the total amount of cell death either following 18h ischemia or 18h ischemia plus 24h reperfusion (Fig 5A and 5B). Interestingly, however, at shorter periods of reperfusion, SENP3 knockdown cells were more susceptible to cell death. Indeed, there were significantly higher levels of cell death in the SENP3 knockdown cells sampled after 18h ischemia and 6h reperfusion compared to cells in which SENP3 levels were not knocked down (Fig 5C). These data suggest that cells with reduced SENP3 die more quickly during reperfusion following ischemic insult, supporting a role for SENP3 in cardiomyocyte survival after ischemia-reperfusion.

## Discussion

Increased understanding of the molecular mechanisms underpinning the pathology of cardiac ischemia and reperfusion is important for the identification of novel drug targets. Here we investigated the molecular changes that occur following a short period of ischemia which can prime cells for reperfusion damage or initiate protection during the subsequent reperfusion phase. In particular, we focused on protein SUMOylation, which has been previously reported to be protective against ischemia/reperfusion damage. Using total homogenate and subcellular fractions from whole hearts subjected to Langendorff perfusion, we observed changes in levels of protein SUMO1-ylation and SUMO2/3-ylation in different subcellular fractions. Furthermore, we detected changes in SUMOylation of individual mitochondrial substrate proteins.

One of the most striking observations in our study was that levels of the SUMO-2/3 selective SUMO protease SENP3 were dramatically reduced in the cytosol during preconditioning, ischemia and ischemia-reperfusion. Moreover, during ischemia, the decrease in cytosolic SENP3 is paralleled by an increase in nuclear SENP3, suggesting that ischemia could cause the relocation of SENP3 from the cytosol to the nucleus. While the overall changes of SENP3 levels were not clearly correlated to changes in overall total protein SUMO2/3-ylation, it is important to note that there are at least six separate members of the SENP family as well as other classes of SUMO proteases [9, 25]. Indeed, SENP1, SENP2 and SENP5 [26–28], as well as SENP3 [14], have each been strongly implicated in cardiac ischemia/reperfusion so it is likely that distinct subsets of SUMOylated proteins are regulated by different SENPs. Therefore, depending on the relative proportions, the impact of modulating levels of one SENP may not be apparent when assessing total global protein SUMOylation but, crucially, it is critical for the regulation of specific substrates.

We have previously demonstrated that stress-induced degradation of SENP3 during ischemia protects HEK293 cells and neurons against reperfusion injury, through promoting SUMOylation of one of its target proteins, Drp1 [21, 29]. In heart tissue ischemia, however, our data suggest a subcellular relocation, rather than loss of SENP3. These differences could reflect cell-type specific mechanisms in controlling the localisation, activity and availability of SENP3 during ischemia. Nonetheless, our data reinforce the concept that SENP3 represents an important stress response protein whose levels and/or localisation are subject to tight control to orchestrate cell survival.

In the cardiac H9C2 cell line ischemia also leads to decreased levels of cytosolic SENP3, which we propose is due to its translocation to the nucleus. We were, however, unable to reliably detect a concomitant increase in nuclear SENP3 in this cell line. SENP3 is predominantly localised in the nucleus with only a small, but physiologically important, fraction present in the cytosol [7, 9]. Thus, detection of an increase mediated by the translocation of a relatively small amount of SENP3 from the cytosol on top of the already high levels of nuclear SENP3 was not technically feasible. Nevertheless, we did confirm that H9C2 cells respond to a short period of ischemia similarly to *ex vivo* heart cells subjected to Langendorff perfusion by reducing cytosolic levels of SENP3. We therefore investigated the effects of SENP3 knockdown and showed increased susceptibility to cell death following I/R in cells with reduced levels of SENP3. Interestingly, however, there was no marked change in the caspase 3 activation in the ischemia stressed SENP3 knockdown cells. More detailed analysis of the temporal profile of caspase 3 activation in H9C2 cells revealed that there was a delayed increase in caspase activity with a peak at 2h of ischemia that diminished after 18h of ischemia. We speculate that this is because levels of ATP are depleted after 18h ischemia and some caspase activation pathways and apoptosis are ATP-dependent processes [30]. Therefore, the cell death we detect at later timepoints by LDH assays is likely due to caspase-independent necroptosis pathways that occur under conditions of ATP depletion [31–33].

Very recently, two other papers have examined the role of SENP3 in cardiac cell survival after I/R [14, 15]. Using *in vivo* ischemia in mice Gao et al. reported no change in total SENP3 levels after 30min of ischemia and increased levels after 3h of reperfusion [14]. Interestingly, cardiac specific SENP3 silencing by intramyocardial injection of siRNA targeting SENP3 decreased infarct size and improved cardiac function after I/R [14]. In addition, Zhang et al. reported an increase of total SENP3 after 2h of ischemia or ischemia/reperfusion in myocardial tissue of mice after ligation of the left coronary artery and after 12h of hypoxia in H9C2 cells [15].

Broadly consistent with these observations, we did not observe changes in total SENP3 after a ‘short-term’ 30min ischemic event, but we did detect changes in the cytosolic SENP3 levels. Moreover, in agreement with Zhang *et al*. [15] the susceptibility of H9C2 cells to I/R-induced death is markedly increased when SENP3 was reduced. Clearly, further work is needed but it seems likely that the roles of SENP3 are influenced by the exact conditions and cell types under investigation. For example, because H9C2 cells are a highly selected and resilient immortalized cell line, longer periods of ischemia are required to induce cell death during the reperfusion period. Similarly, the vulnerability of different cell types within the heart and the consequences of SENP3 loss may vary from the effect observed specifically in cardiomyocytes or H9C2 cells. Indeed, reducing SENP3 promotes HEK293 and neuronal survival after I/R [21, 29], suggesting that the pro-survival role of SENP3 observed here may result from cell type-specific differences in the response to ischemia.

Our data suggest a protective role for SENP3 in whole heart, because preconditioning prior to ischemia reduced the extent of SENP3 loss from the cytosol compared to ischemia alone. Since preconditioning potently protects cells against I/R injury [34], and loss of SENP3 in H9C2 cells promotes cell death upon I/R, these data suggest that the protective effects of preconditioning could, at least in part, be due to the effect of preconditioning in limiting SENP3 loss from the cytosol.

SENP3 deconjugates SUMO2/3 from a wide variety of substrates, the majority of which have not yet been identified [7, 9]. Our previous work demonstrated a role for SENP3 in the response to ischemia through modulation of SUMOylation of its target Drp1 [21, 29], so it is possible that Drp1 is the major SENP3 target mediating its effects in cardiomyocytes. However, our initial experiments suggest that the changes we observed in SENP3 localisation during PCI, ischemia and I/R are not mirrored by corresponding changes in Drp1 localisation at mitochondria, which is a direct outcome of Drp1 SUMOylation [21, 29, 35]. Thus, it seems likely that other, as yet unidentified extranuclear SENP3 targets may be mediating its protective effect on cardiomyocytes. Indeed, it is of note that we observed alterations in specific mitochondria-associated SUMO substrates during PCI, ischemia and I/R, suggesting an altered SUMOylation profile of a restricted number of cytosolic or mitochondrial substrates may coordinate cardiomyocyte survival after I/R.

Together, our data demonstrate that in whole perfused hearts short-term ischemia leads to a robust drop of cytosolic SENP3, while preconditioning attenuates this decrease. Similarly, in H9C2 cells a short period of ischemia reduces cytosolic SENP3 whereas SENP3 levels are restored following longer periods of ischemia. Moreover, in H9C2 cells SENP3 knockdown reduces survival following I/R. Thus, we suggest that SENP3 plays a cardioprotective role during ischemia-reperfusion and promoting SENP3 levels in susceptible individuals, or maintaining the levels of SENP3 after ischemic infarct, may represent novel therapeutic strategies aimed at promoting cell survival and heart function after cardiac ischemia.

## Supporting information

S1 Fig Perfusion protocols used for simulating global myocardial ischemia (Isc), preconditioning before global ischemia (PCI) and ischemia followed by reperfusion (I/R). (TIF)
