# Supplementary figures and images for: Protective role of the deSUMOylating enzyme SENP3 in myocardial ischemia-reperfusion injury
Source: PLoS One. 2019 Apr 11;14(4):e0213331. doi: 10.1371/journal.pone.0213331 (PMC6459529; doi:10.1371/journal.pone.0213331)

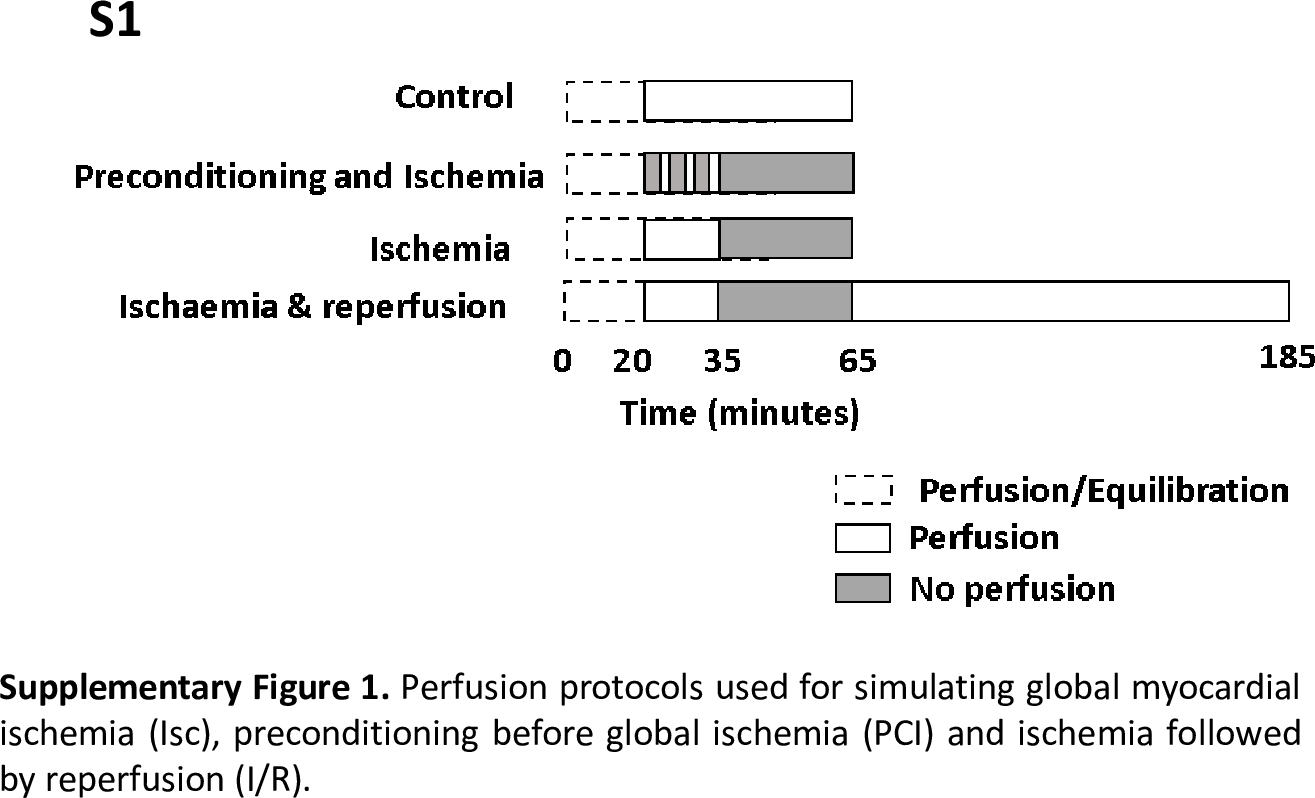

Supplement: S1 Fig — (TIF) [file pone.0213331.s001.tif]
